# A Computational Mechanism for Seeing Dynamic Deformation

**DOI:** 10.1523/ENEURO.0278-19.2020

**Published:** 2020-04-07

**Authors:** Takahiro Kawabe, Masataka Sawayama

**Affiliations:** NTT Communication Science Laboratories, Nippon Telegraph and Telephone Corporation, Atsugi, 243-0198, Japan

**Keywords:** computational model, deformation, MT, vision

## Abstract

Human observers perceptually discriminate the dynamic deformation of materials in the real world. However, the psychophysical and neural mechanisms responsible for the perception of dynamic deformation have not been fully elucidated. By using a deforming bar as the stimulus, we showed that the spatial frequency of deformation was a critical determinant of deformation perception. Simulating the response of direction-selective units (i.e., MT pattern motion cells) to stimuli, we found that the perception of dynamic deformation was well explained by assuming a higher-order mechanism monitoring the spatial pattern of direction responses. Our model with the higher-order mechanism also successfully explained the appearance of a visual illusion wherein a static bar apparently deforms against a tilted drifting grating. In particular, it was the lower spatial frequencies in this pattern that strongly contributed to the deformation perception. Finally, by manipulating the luminance of the static bar, we observed that the mechanism for the illusory deformation was more sensitive to luminance than contrast cues.

## Significance Statement

From the psychophysical and computational points of view, the present study tried to answer the question, “how do human observers see deformation?”. In the psychophysical experiment, we used a clip wherein a bar dynamically deformed. We also tested the illusory deformation of a bar, which was caused by tilted drifting grating, because it was unclear whether the illusory deformation could be described by our model. In the computational analysis, to explain psychophysical data for deformation perception, it was necessary to assume an additional unit monitoring the spatial pattern of direction responses of MT cells that were sensitive to local image motion.

## Introduction

Materials in the real world are often non-rigid. The material non-rigidity dynamically produces the deformations of contours and textures in the retinal images. The dynamic deformation of retinal images is a rich source of visual information allowing the visual system to assess material properties in the real world. For example, from the dynamic deformation of retinal images, human observers can recognize transparent liquid (Kawabe et al., 2015), transparent gas (Kawabe and Kogovšek, 2017), the elasticity and/or stiffness of materials (Masuda et al., 2013, 2015; Paulun et al., 2017; Schmidt et al., 2017), the stiffness of fabrics (Bi and Xiao, 2016; Bi et al., 2018, 2019), biological motion (Johansson, 1973; Blake and Shiffrar, 2007; Kawabe, 2017), and more.

Importantly, however, the visual mechanism for detecting dynamic deformation itself has not been thoroughly examined. Previous studies have reported that observers reported dynamic deformation when local motion integration did not provide evidence for rigid motion (Nakayama and Silverman, 1988a,b) at a layer-represented level (Weiss and Adelson, 2000). Although successfully addressing the stimulus condition in which rigid motion perception was violated, these previous studies have not mentioned the conditions to cause the perception of dynamic deformation. Although some studies have proposed the computational model for the detection of two-dimensional motion patterns containing shearing and/or rotating motion (Sachtler and Zaidi, 1995; Zhang et al., 1993), the model is not directly considered as the explanation of deformation perception because, as shown in a previous study (Nakayama and Silverman, 1988a), a shearing motion pattern does not always cause deformation perception. Nakayama and Silverman (1988a) showed that dynamic contour deformation with higher deformation frequency did not produce deformation perception but caused the rigid movement of wavy patterns, indicating that the detection of the shearing motion itself does not always lead to shearing deformation perception. No previous computational model exactly accounts for the dependency of deformation perception on the spatial frequency of deformation. Hence, additional examinations are necessary to fully understand mechanisms for deformation perception in human observers. Moreover, it remained unclear what visual information could be effective in generating the representation of dynamic deformation. Jain and Zaidi (2011) have shown that motion is important information for discerning the shape of non-rigidly deforming objects. The present study thus focuses on how motion information contributes to the perception of dynamic deformation.

The purpose of this study was to psychophysically and computationally specify the mechanism that underlies the perception of dynamic deformation. In experiment 1, using stimuli with a physically deforming bar we show that the spatial frequency of deformation is an important factor to phenomenally determine deformation perception. By simulating both spatiotemporal energy responses at the V1 level and the responses of direction-selective units (i.e., MT pattern motion cells; Simoncelli and Heeger, 1998; Perrone and Krauzlis, 2008, 2014), we show that not spatiotemporal motion energy but the spatial pattern of the responses of the direction-selective units consistently explains observers’ reports for the perception of dynamic deformation. In experiment 2, we first examine a visual illusion in which a static bar with solid edges apparently deforms against a slightly tilted drifting grating. We report that both the orientation and spatial frequency of the background grating are critical to the illusory perception of deformation. From the observation, it is plausible to assume that moiré patterns (Oster, 1965; Spillmann, 1993; Wade, 2007), which are generated between the bar’s edge and background grating, produce motion signals that are related to the apparent deformation. However, it is still unclear whether the dependence of the deformation appearance on both orientation and spatial frequency can be explained by the spatial pattern of the responses of direction-selective units. We again analyze the spatial pattern of the direction-selective unit responses to the illusion display and examine whether they again predict the observers’ report of deformation in the illusory deformation. In experiment 3, we show that the perception of illusory dynamic deformation is attenuated when the average luminance of background grating is equivalent to the luminance of a bar. We then discuss how the perception of dynamic deformation is determined on the basis of the output of a high-level mechanism monitoring the spatial patterns of responses of direction-selective units.

## Materials and Methods

### Experiment 1

#### Observers

Seven people (five females and two males) participated in this experiment. All observers in this study reported having normal or corrected-to-normal visual acuity. They were recruited from outside the laboratory and received payment for their participation. Ethical approval for this study was obtained from the ethics committee at Nippon Telegraph and Telephone Corporation (approval number: H28-008 by NTT Communication Science Laboratories Ethical Committee). The experiments were conducted according to principles that have their origin in the Helsinki Declaration. Written, informed consent was obtained from all observers in this study.

#### Apparatus

Stimuli were presented on a 21-inch iMac (Apple Inc. USA) with a resolution of 1280 × 720 pixels and a refresh rate of 60 Hz. A colorimeter (Bm-5A, Topcon) was used to measure the luminance emitted from the display. A computer (iMac, Apple Inc.) controlled stimulus presentation, and data were collected with PsychoPy v1.83 (Peirce, 2007, 2009).

#### Stimuli

**Figure 1.**
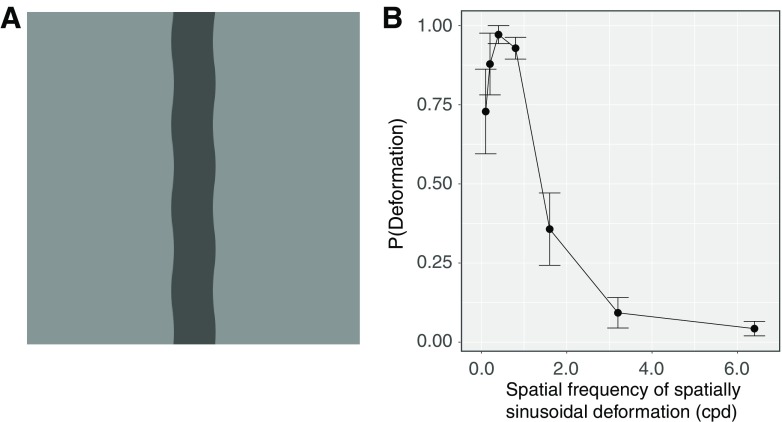
***A***, A snapshot of a stimulus clip as used in experiment 1 (Extended Data Movie 1). ***B***, Experiment 1 results. Error bars denote SEM (N = 7).

In the stimulus (Fig. 1*A*; Extended Data Movie 1), the edge of a vertical bar (0.6° width × 5.0° height) was horizontally deformed at one of the following seven spatial frequencies [0.1, 0.2, 0.4, 0.8, 1.6, 3.2, and 6.4 cycles per degree (cpd)]. We chose the range of spatial frequency of deformation to cover the range tested in the previous study (Nakayama and Silverman, 1988a). The amplitude was kept constant at 0.04 cpd. With each stimulus, upward or downward drifting was randomly given to the modulation. The modulation temporal frequency was 1 Hz. The luminance of the bar was randomly chosen as one of two levels (38 and 114 cd/m^2^). The luminance of the background was 76 cd/m^2^.

Movie 1. Some examples of stimulus video clips as used in Experiment 1. 10.1523/ENEURO.0278-19.2020.video.1

#### Procedure

Each observer was tested in a lit chamber. The observers sat 102 cm from the display. With each trial, a stimulus clip having a deforming bar was presented for 3 s. After the disappearance of the clip, a two-dimensional white noise pattern (with each cell subtending 0.16° × 0.16°) was presented until the observer’s response. The task of the observers was to judge whether a bar dynamically deformed or not. The judgment was delivered by pressing one of the assigned keys. Each observer had two sessions, each consisting of seven spatial frequencies of the modulation × 10 repetitions. Within each session the order of trials was pseudo-randomized. Thus, each observer had 140 trials in total. It took ∼20 min for each observer to complete all sessions.

### Experiment 2

#### Observers

Twelve people (10 females and two males) participated in this experiment. Their mean age was 38.2 (SD, 7.63). Although seven of them also already participated in the previous experiment, none was aware of the specific purpose of the experiment because there was no preliminary explanation or debriefing provided.

#### Apparatus

Apparatus was identical to that used in experiment 1.

#### Stimuli

As shown in Figure 4*A* (and Extended Data Movies 2, 3, 4), a vertical bar (0.6° wide × 5.0° high) was presented in front of a drifting grating. For each stimulus, the luminance of the bar was randomly chosen from two levels (37 and 112 cd/m^2^). The orientation of the background grating was selected from the following seven levels (0.5°, 1°, 2°, 4°, 8°, 12°, and 16°). The spatial frequency was selected from the following 3 levels (6.4, 12.9, and 25.8 cpd). Drift temporal frequency was kept constant at 1 Hz. The drift direction was randomly determined. The luminance contrast of the grating was set at 0.75, and thus the luminance level of the grating ranged between 37 and 112 cd/m^2^. The drifting grating was windowed by a horizontal Gaussian envelope with a SD of 0.62°.

Movie 2. Some examples of stimulus video clips as used in the 25.8 cpd condition of Experiment 2. 10.1523/ENEURO.0278-19.2020.video.2

Movie 3. Some examples of stimulus video clips as used in the 12.9 cpd condition of Experiment 2. 10.1523/ENEURO.0278-19.2020.video.3

Movie 4. Some examples of stimulus video clips as used in the 6.4 cpd condition of Experiment 2. 10.1523/ENEURO.0278-19.2020.video.4

#### Procedure

Procedure was identical to that in the previous experiment except for the following. With each trial, a stimulus clip having a static bar and drifting grating was presented for 3 s. After the disappearance of the clip, visual white noise (each cell subtending 0.16° × 0.16°) was presented until the observer’s response. The task of the observers was to judge whether the static bar dynamically deformed or not. Each observer had four sessions, each consisting of three spatial frequencies × seven orientations × five repetitions. Within each session the order of trials was pseudo-randomized. Thus, each observer had 420 trials in total. It took 30–40 min for each observer to complete all four sessions.

### Experiment 3

#### Observers

Twelve people who had participated in experiment 1 again participated in this experiment. Still, none was aware of the specific purpose of the experiment.

#### Apparatus

Apparatus was identical to that used in experiment 1.

#### Stimuli

Stimuli were identical to those used in experiment 1 except for the following. The background grating orientation was 1° or 16°, which respectively produced strong deformation and non-deformation responses in experiment 1. The grating spatial frequency was kept constant at 12.9 cpd. As shown in Extended Data Movie 5, the luminance of the bar was randomly chosen from the nine levels (0.0, 17.5, 37, 58, 76, 95, 112, 132, and 148 cd/m^2^ wherein 76 cd/m^2^ was the luminance of a neutral gray level).

Movie 5. Some examples of stimulus video clips as used in Experiment 3. 10.1523/ENEURO.0278-19.2020.video.5

#### Procedure

Procedure was identical to that used in experiment 1 except for the following. Each observer had four sessions, each consisting of two levels of grating orientation × nine luminance levels of the bar × five repetitions. Within each session the order of trials was pseudo-randomized. Thus, each observer performed 360 trials in total. It took ∼20 min for each observer to complete all of both sessions.

### Simulation of MT responses

**Figure 2.**
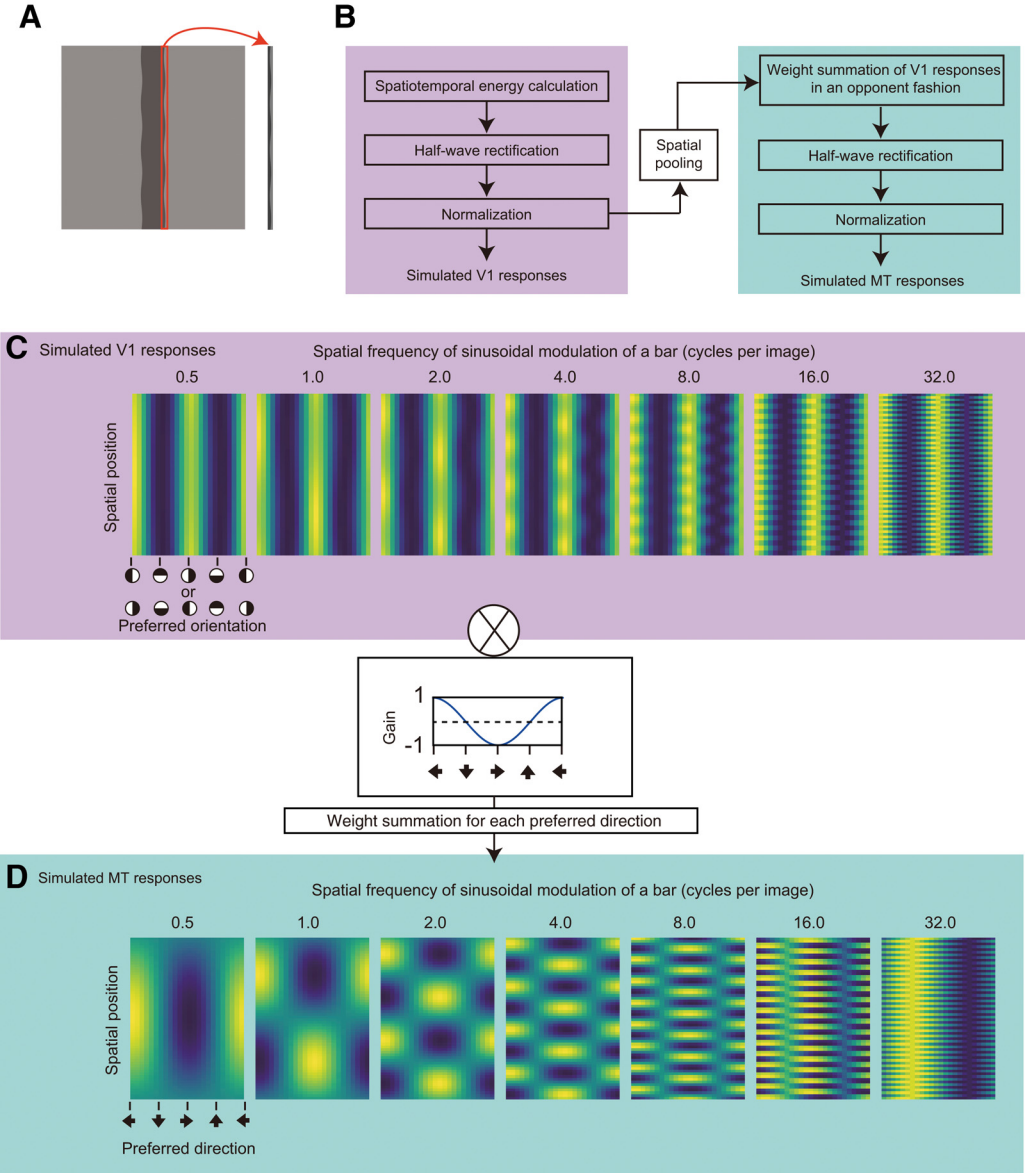
***A***, Extracted area for simulation. The red-bound area was used to simulate the response of the direction-selective units. ***B***, A pipeline of our model. ***C***, Simulated spatiotemporal motion energy for the stimuli of experiment 1. In this panel, the range of each density plot is normalized between 0 and 1. Raw values were used for further analysis. ***D***, Simulated responses of direction-selective units for the stimuli of experiment 1. In this panel, the range of each density plot is normalized between 0 and 1. Raw values were used for further analysis.

For the stimuli that were used in experiments 1 and 2, we simulated the responses of direction-selective units on the basis of previous studies (Simoncelli and Heeger, 1998; Mante and Carandini, 2005; Perrone and Krauzlis, 2008, 2014; Nishimoto and Gallant, 2011). We extracted a stimulus area near the right vertical edge of the bar that was presented against a background (Fig. 2*A*) and simulated the responses of the direction-selective units to the area.

#### Spatial parameters of spatiotemporal energy detection

In experiment 1, we set the width of the extracted area for analysis at 0.08° because the amplitude of contour deformation applied to the bar was 0.04°. In experiment 2, based on the spatial frequency of the background grating, we changed the width of the extracted area for analysis (0.16°, 0.08°, and 0.04° for 6.4, 12.9, and 24.8 cpd conditions). The height of the extracted area was constant at 4.97°. The extracted area was first analyzed by a set of spatiotemporal filters. In experiment 1, width, height, and spatial wavelength of the spatiotemporal filters were all 0.08°. In experiment 2, width, height, and spatial wavelength of the spatiotemporal filters were also consistent with the width of the extracted area, that is, 0.16°, 0.0 °, and 0.04° for 6.4, 12.9, and 24.8 cpd conditions, respectively. The filters with different phases (0π or 1.0π) were independently applied to stimuli and the outputs of the filters with different phases were later summed after the half-wave rectification and normalization described below. The number of filter orientations was 24 (i.e., 15° steps). The position of the filters did not spatially overlap. In experiment 1, 64 filters covered the extracted area. In experiment 2, 32, 64, and 128 filters covered the extracted area for 6.4, 12.9, and 24.8 cpd conditions, respectively.

#### Temporal parameters of spatiotemporal energy detection

In both experiments, the temporal size of the filter was 6 frames (for ∼100 ms) and the temporal frequency was fixed at 0.4 Hz. The combination of temporal and spatial frequencies of the filter was optimized to the stimulus speed in the stimuli. We adopted the temporal properties because we wanted to extract a one-way modulation of the moiré pattern.

#### Rectification, normalization, and spatial pooling

The responses of the filters were half-wave rectified and normalized as reported in the previous study (Simoncelli and Heeger, 1998). In the calculation of normalization, as suggested by the previous study (Simoncelli and Heeger, 1998), the half-wave rectified output, which was multiplied by the maximum attainable response constant, was divided by the summed output of half-wave rectified responses across orientations and the semi-saturation constant. The normalized responses are considered as the spatiotemporal energy. The calculated motion energy is plotted in Figures 2*C*, 5*B* for experiment 1 and experiment 2, respectively. The normalized responses were spatially pooled among four adjacent filters, yielding 29, 61, and 125 responses. A Gaussian filter, which was centered on the pooling range, was applied to the pooling. The SD of the Gaussian filter was 1.2.

#### Calculation of direction-selective responses

The pooled responses were filtered by a direction-tuned filter (Perrone and Krauzlis, 2008, 2014) that tuned to the motion direction using a cosine function. That is, at this level, the rectified and normalized outputs of the spatiotemporal energy filters were summed with weightings in an opponent fashion. The direction-tuned filters had 24 preferred directions (i.e., 15° steps). The filtered responses were half-wave rectified and then normalized as in a previous study (Simoncelli and Heeger, 1998). The constants were just identical to those as used in the previous study (Simoncelli and Heeger, 1998). This consequently yielded the responses of direction-selective units as functions of the preferred direction of units and spatial position, as shown in Figure 2*D* for experiment 1 and Figure 5*C* for experiment 2.

### Properties of units monitoring the spatial pattern of direction responses

**Figure 3.**
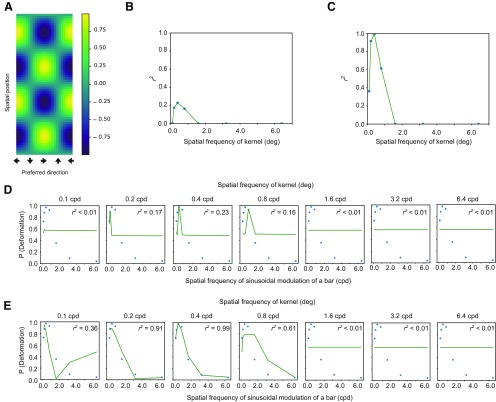
***A***, An example of the kernel which was employed here. ***B***, ***C***, The vertical axis denotes the coefficient determination (r2) for the fitting of an exponential function to the proportion of trials with deformation reports as a function of NCC, and the horizontal axis denotes the spatial frequency of modulation of a kernel. The panel B is for spatiotemporal motion energy, and the panel ***C*** is for the response of direction-selective units. ***D***, ***E***, The psychophysical data of deformation reports (markers) are jointly plotted with the fitted values (lines) as a function of the spatial frequency of sinusoidal deformation. The panel D is for spatiotemporal motion energy, and the panel ***E*** is for the response of direction-selective units.

The following analysis was conducted to calculate the normalized cross-correlation (NCC) between spatiotemporal motion energy (or the response of direction-selective units) and the kernel of assumed higher-order units that are possibly sensitive to the spatial variation of spatiotemporal motion energy or the responses of direction-selective units. The kernel was defined by the product of the spatially sinusoidal pattern S and the directionally sinusoidal pattern D (Fig. 3*A*). S was defined by the following formula,

(1) S(x)= sin(2fx+ϕ),

where *f* denotes spatial frequency, *ϕ* denotes phase, and x denotes spatial position. D was defined by the following formula,

(2) D(θ)= cos(2π(θ−α)),

where *θ* denotes the motion direction and ranges from 0 to 2π, and α denotes the preferred direction of the kernel. Here, α was set to 0° (leftward direction) on the basis of the spatial pattern of the direction-selective units as shown in Figure 2*D*. Thus, a kernel K was defined as the product of S and D,

(3) K(x,θ)=S(x)D(θ).

To see how the kernel (Fig. 3*A*) matched the spatiotemporal motion energy (Fig. 2*C*) or the response of direction-selective units (Fig. 2*D*), we calculated the NCC between them. The NCC was calculated by the following formula:

(4) $$NCC=\frac{\sum \sum (K-\mu_{K})(I-\mu_{I})}{\sqrt{\sum \sum (K-\mu_{K})}\sqrt{\sum \sum (I-\mu_{I})}},$$

where *K* is the kernel, *I* is the spatiotemporal motion energy (or the pattern of direction-selective units), and *μ_K_* and *μ_I_* are the mean of *K* and *I*, respectively. This NCC is often called as the zero-mean NCC. The f of (1) took one of the following levels: 0.1, 0.2, 0.4, 0.8, 1.6, 3,2 and 6.4 cpd. The ϕ was tested in 32 steps (each step = 0.0625 π), and the maximum value among the 32 outcomes based on the 32 steps was considered to be the NCC of the kernel.

## Results

### Experiment 1

The purpose of experiment 1 was to specify psychophysical parameters to cause the perception of dynamic deformation and look into a possible relationship between the psychophysical data and the simulation data of neural responses such as V1 motion energy and MT pattern motion cells. Although a previous study (Nakayama and Silverman, 1988a) investigated amplitude thresholds at which dynamic deformation disappeared when a sinusoidally modulated line translated, no studies have directly examined which spatial frequencies of deformation produced the largest effect on deformation perception. Here we asked the observers to report whether the bar (which was actually deformed in the display) was seen as deforming, and examined the relationship between the observers’ responses and the spatial frequency of deformation.

Figure 1*B* shows the proportion of trials wherein the observers reported the bar as dynamically deforming. By using the proportion, we conducted a repeated-measures one-way analysis of variance with the spatial frequency of modulation as a within-subject factor. The main effect was significant [*F*_(6,36)_ = 38.358, *p *<* *0.0001, *η^2^_p_* = 0.86]. Multiple comparison showed that the proportions in the 0.1, 0.2, 0.4, and 0.8 cpd conditions were significantly higher than the proportions in the 1.6, 3.2, and 6.4 cpd conditions (*p *<* *0.05). The results showed that the lower spatial frequency of deformation contributed to the perception of dynamic deformation more strongly than the higher spatial frequency of deformation, consistent with the previous study (Nakayama and Silverman, 1988a) showing that the amplitude thresholds at which dynamic deformation was abolished increased for lower spatial frequencies of physical deformation of a line. When the spatial frequency of deformation was high, some observers reported that they saw a rigid translation of wavy patterns along the edge of a bar; this was also consistent with the previous study (Nakayama and Silverman, 1988a).

### Simulation of MT responses

To specify the mechanism for seeing dynamic deformation in our stimuli, we decided to check the relationship between the perception of dynamic deformation and the simulated responses of units at the V1 and MT levels. On the basis of the previous literature (Simoncelli and Heeger, 1998; Mante and Carandini, 2005; Perrone and Krauzlis, 2008, 2014; Nishimoto and Gallant, 2011), we constructed the simulation by employing a standard procedure with the following steps (see also Fig. 2*B* and Materials and Methods for details): (1) convolving stimuli with spatiotemporal filters to get spatiotemporal motion energy; (2) half-wave rectification; (3) divisive normalization, at this stage, spatiotemporal energy of stimuli was obtained; (4) spatial pooling with a spatially gaussian window; (5) weighted summation of spatiotemporal energy in an opponent fashion; (6) half-wave rectification; and (7) divisive normalization, at this stage, the response of a direction-selective unit was obtained.

Figure 2*C* shows the spatiotemporal motion energy as functions of the preferred spatiotemporal orientation and spatial position, for each condition of the spatial frequency of sinusoidal deformation. Figure 2*D* shows the responses of direction-selective units as functions of the preferred direction of the units and spatial position, for each condition of the spatial frequency of sinusoidal deformation. For both spatiotemporal energy and the responses of direction-selective units, the spatial pattern got finer as the spatial frequency of deformation increased. The spatiotemporal motion energy was high at the vertical orientations consistently across space. On the other hand, the response of direction-selective units was high at the leftward and rightward but in a spatially alternating manner.

### Properties of units monitoring the spatial pattern of direction responses

Based on the simulation, we next examined whether the psychophysical results could be well accounted for by spatiotemporal motion energy and/or the responses of direction-selective units. The brain would determine if the input signal came from deformation or not, based on the output of the higher-order unit that is sensitive to the spatial pattern of spatiotemporal motion energy and/or the responses of direction-selective units. To test this prediction, we conducted a pattern-matching analysis using a kernel as shown in Figure 3*A*. From the results of the simulation of the spatiotemporal motion energy (Fig. 2*C*) and direction-selective units (Fig. 2*D*), it was plausible to assume that the higher-order units could tune to spatially sinusoidal modulation of motion direction as shown in Figure 3*A*. We call the template pattern having the spatially sinusoidal modulation “a kernel.” To assess the similarity between the kernel and each spatial pattern of spatiotemporal motion energy and the spatial pattern of the response of direction-selective unit, manipulating the spatial frequency of the modulation in the kernel, we calculated the NCC between the kernel and the spatial pattern of the spatiotemporal motion energy or the responses of direction-selective units. We assumed that the calculated NCC could account for the psychophysical data for deformation perception. It was thus expected that the NCC would well explain the psychophysical data when the kernel had a high correlation with the simulated neural responses that effectively contribute to the deformation perception. We calculated NCCs between a kernel with one of the pre-determined spatial frequency of modulation and the spatial pattern of the spatiotemporal motion energy (Fig. 2*C*) and/or the simulated responses of direction-selective units (Fig. 2*D*).

Next, we fitted an exponential function to the psychophysical data as a function of the NCCs. The coefficient of determination (*r*^2^) of the fitting is plotted in Figure 3*B* for the spatiotemporal motion energy and in Figure 3*C* for the responses of direction-selective units. The psychophysical data (markers) and fitted values (lines) are jointly plotted in Figure 3*D* for the spatiotemporal motion energy and in Figure 3*E* for the response of direction-selective units as a function of the spatial frequency of sinusoidal deformation. For both the spatiotemporal motion energy and the responses of direction-selective units, the lower bands of the spatial frequency of the modulation in the kernel showed the highest coefficient of determination. The highest coefficient of determination implies that for the brain the information at the lower spatial frequency range reliably contributes to the estimation of dynamic deformation. Moreover, the coefficient of determination was higher for the response of direction-selective units than for the spatiotemporal motion energy.

The results suggest that the perception of bar deformation is likely mediated by the higher-order unit monitoring the spatial pattern of the responses of the direction-selective units. Moreover, the observers’ reports for deformation perception seem tuned to the lower spatial frequency of the responses of direction-selective units. The results are well consistent with the previous study showing that the spatial frequency of image deformation determines the appearance of image deformations (Kawabe et al., 2015). The higher coefficient of determination for the response of direction-selective units than the spatiotemporal motion energy indicates that deformation perception is based on the direction responses at the MT area rather than the spatiotemporal motion energy at the V1 area.

The simulated response of the V1 cell was spatially sinusoidal but a little bit noisy. The noisiness of the response might come from that the orientation of a stimulus edge was near-vertical. Thus, there was a possibility that most of the V1 cells that were sensitive to the vertical orientation captured the signals of the edges, making the baseline of their activity non-zero along the edge. The high baseline possibly attenuated the sinusoidal pattern of the V1 responses. On the other hand, the response of direction-selective units does not depend strongly on the stimulus orientation because the MT cells solve the aperture problem. The solution of the aperture problem possibly made the simulated response of the direction-selective units less noisy than the simulated response of the V1 cells.

When the spatial frequency of the sinusoidal modulation of a bar was high, the simulated responses of direction-selective units showed preferences for downward motion (Fig. 2*D*). This pattern of responses is consistent with the psychophysical data in a previous study (Nakayama and Silverman, 1988a), which showed that the sinusoidal modulation of a line perceptually resulted in unidirectional translation when it had a high spatial frequency of modulation. The results of our simulations indicate that the model we employed could precisely capture the properties of human motion perception with stimuli containing dynamic deformation.

### Experiment 2

**Figure 4.**
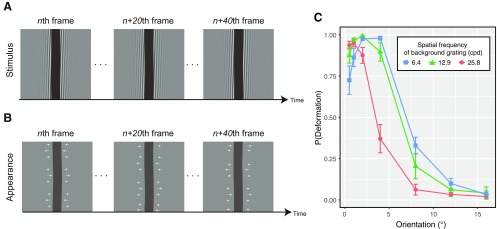
***A***, Several snapshots of stimuli as used in experiment 2. ***B***, Schematic explanations of the appearance of the deformation illusion of a bar on the basis of background drifting grating. ***C***, Proportions of deformation reports as a function of the orientation of background drifting grating for each spatial frequency condition of the background grating.

The purpose of this experiment was to confirm whether illusory deformation perception that is induced by moiré pattern (Fig. 4*A*,*B*) could also be explained by the activities of higher-order units that are tuned to the spatial pattern of responses of direction-selective units.

**Figure 5.**
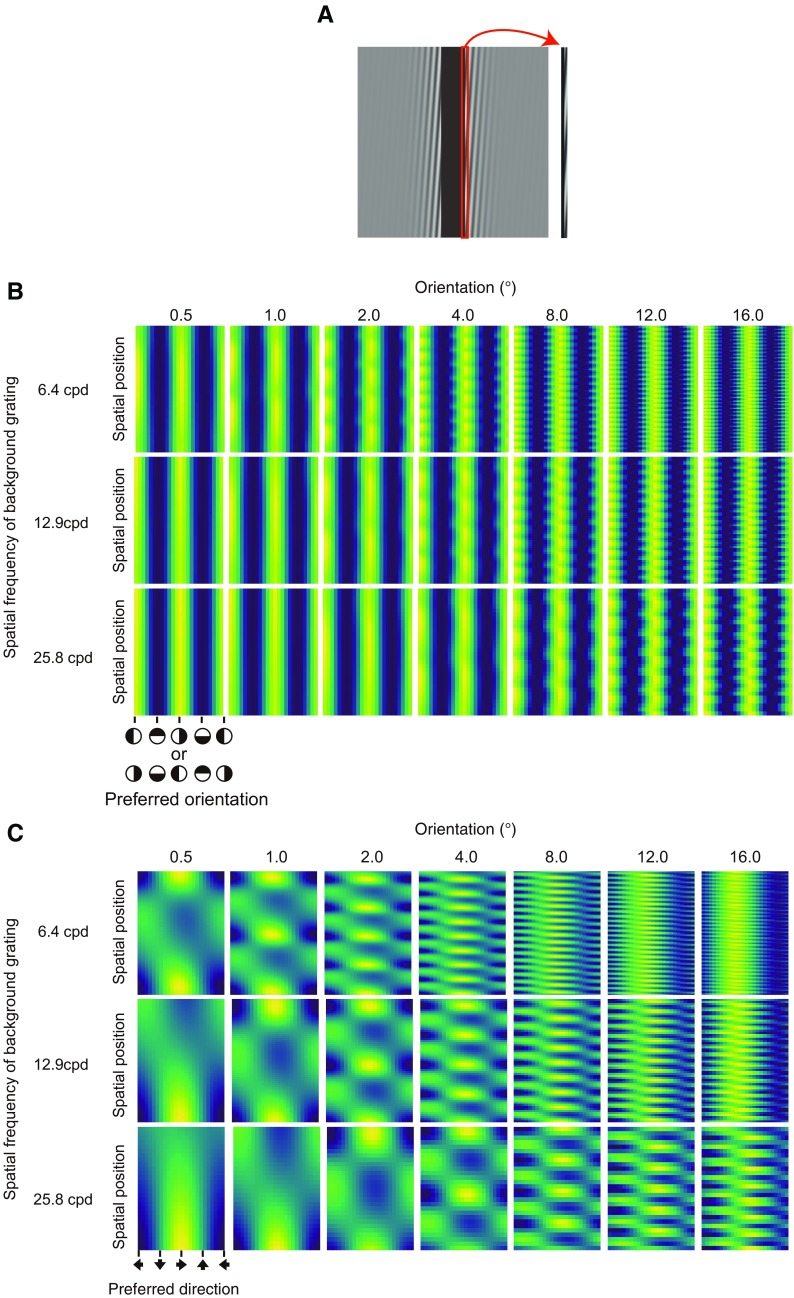
***A***, Extracted area for simulation. ***B***, Simulated spatiotemporal motion energy for the stimuli of experiment 1. In this panel, the range of each density plot is normalized between 0 and 1. Raw values were used for further analysis. ***C***, Simulated responses of direction-selective units for the stimuli of experiment 1. In this panel, the range of each density plot is normalized between 0 and 1. Raw values were used for further analysis.

We calculated the proportion of trials in which the observer reported the dynamic deformation of the bar and plotted them as a function of grating orientation for each spatial frequency condition in Figure 4*C*. We conducted a two-way repeated-measures analysis of variance with grating orientation and grating spatial frequency as within-subject factors. The main effect of the grating spatial frequency was significant [*F*_(2,22)_ = 27.440, *p *<* *0.0001, *η^2^_p_* = 0.72]. The main effect of the grating orientation was also significant [*F*_(2,22)_ = 255.772, *p *<* *0.0001, *η^2^_p_* = 0.96]. Interaction between the two factors was significant [*F*_(12,132)_ = 15.362, *p *<* *0.0001, *η^2^_p_* = 0.58). The results showed that the illusory deformation occurred when the background drifting grating had smaller tilts away from vertical. At the same time, there was a significant interaction between orientation and spatial frequency of the background grifting grating. The significant interaction possibly comes from the peak shift of the deformation reports which occurred depending on the spatial frequency of background grating. As the spatial frequency of background grating decreased, the peak of deformation reports occurred at the shallower orientation of the grating.

**Figure 6.**
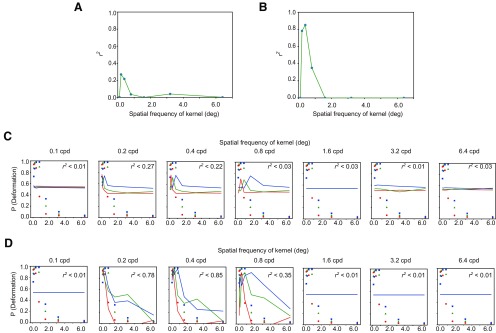
***A***, ***B***, The vertical axis denotes the coefficient determination (r2) for the fitting of an exponential function to the proportion of trials with deformation reports as a function of NCC, and the horizontal axis denotes the spatial frequency of modulation of a kernel. The panel ***A*** is for spatiotemporal motion energy, and the panel B is for the response of direction-selective units. ***C***, ***D***, The psychophysical data of deformation reports (markers) are jointly plotted with the fitted values (lines) as a function of the spatial frequency of sinusoidal deformation. The panel ***C*** is for spatiotemporal motion energy, and the panel D is for the response of direction-selective units.

We surmised that the peak shift might come from the change in the activity of the higher-order units that were sensitive to the spatial pattern of the responses of direction-selective units, and hence decided to simulate the responses of direction-selective units for the stimuli as used in this experiment. In a similar way to the previous analysis, by manipulating the spatial frequency of the kernel (Fig. 3*A*) we calculated the NCC for each stimulus and fitted an exponential function to the psychophysical data as a function of the NCCs. The coefficients of determination (*r*^2^) of the fitting are shown in Figure 6*A* for the spatiotemporal motion energy and in Figure 6*B* for the response of direction-selective units. As in the previous analysis, *r*^2^ peaked at the lower spatial frequency bands of the spatial pattern of direction responses. The psychophysical data and fitted values are jointly plotted in Figure 6*C* for the spatiotemporal motion energy and in Figure 6*D* for the response of direction-selective units as a function of the spatial frequency of sinusoidal modulation. Similar to the results of experiment 1, the coefficient of determination was higher for the response of direction-selective units than for the spatiotemporal motion energy.

The results indicate that similar to the stimuli that contained the actual deformation of a bar, the brain uses the output of the higher-order units that are sensitive to the spatial pattern of responses of direction-selective units in determining illusory deformation perception. Moreover, consistent with the results of experiment 1, the response of direction-selective units at the MT area possibly more strongly contributes to the illusory deformation perception than the spatiotemporal motion energy.

### Experiment 3

The purpose of this experiment was to additionally confirm whether the deformation perception could be obtained on the basis of the contrast-based moiré pattern. In experiment 2, we used dark and bright bars against a background drifting grating, so the moiré pattern generated between the bar and the background grating was always defined by luminance. By testing the condition with the bar luminance at a neutral gray (Extended Data Movie 5), we investigated whether the contrast-based moiré pattern also contributed to the perception of dynamic deformation. If the deformation perception was operated by the mechanism that had a function equivalent to the direction-selective units, because the unit was assumed to have selectivity to luminance, no strong influence of contrast-based moiré pattern would be observed.

**Figure 7.**
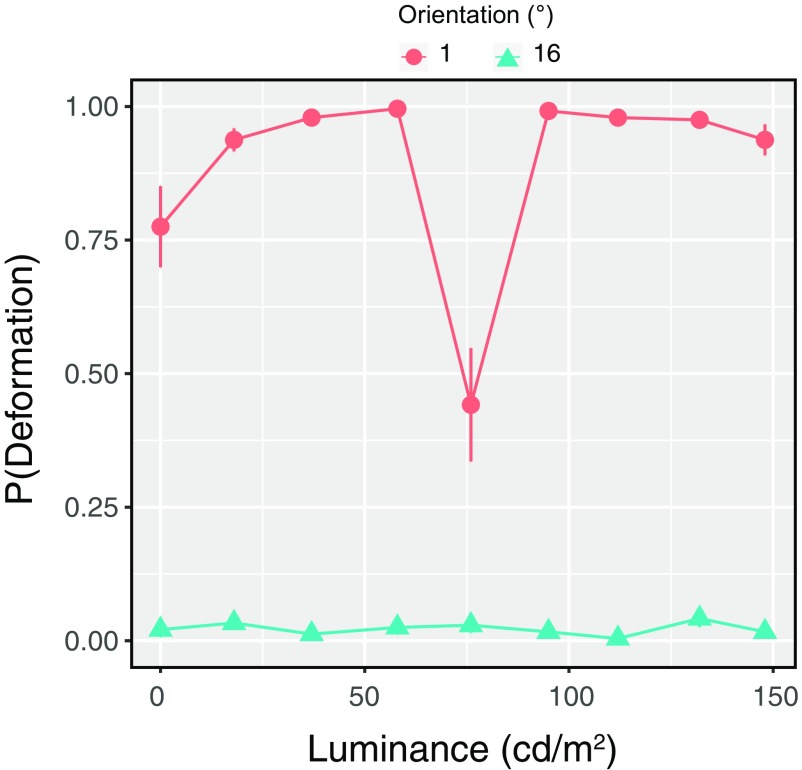
Experiment 3 results. Proportions of deformation reports as a function of the luminance of a bar in stimuli. Error bars denote ±1 SEM for each of the orientation conditions of background drifting grating.

In Figure 7, the proportion of trials with reports of bar deformation is plotted for each background orientation condition as a function of the luminance of the bar. As in experiment 1, the deformation perception was more often reported with a grating orientation of 1° than 16°. Interestingly, the proportion suddenly dropped when the luminance of the bar was set at the level of neutral gray. By using the proportion, we conducted a two-way repeated-measures analysis of variance with the bar luminance and background grating orientation as within-subject factors. The main effect of the bar luminance was significant [*F*_(8,88)_ = 16.36, *p *<* *0.0001, *η^2^_p_* = 0.60]. The main effect of the background grating orientation was also significant [*F*_(1,11)_ = 1463.35, *p *<* *0.0001, *η^2^_p_* = 0.99]. Interaction between the two factors was also significant [*F*_(8,88)_ = 17.763, *p *<* *0.0001, *η^2^_p_* = 0.62]. The simple main effect based on the significant interaction showed that the proportion for 76 cd/m^2^ was significantly lower than the proportions for other luminance conditions when the background orientation was 1° (*p *<* *0.05). The results showed that the deformation perception was attenuated when the bar luminance was neutral gray, suggesting that the mechanism for the deformation perception in our illusion is most sensitive when there is a difference in overall luminance between a bar and its background. Moreover, the illusory deformation was still reported even when the luminance of the bar itself was outside of the luminance contrast range of the background grating. The results suggest that the deformation illusion triggered by moiré patterns occurs with a flexible relationship between the bar and the background grating.

## Discussion

This study investigated the mechanism responsible for deformation perception by human observers. Our data suggest that the brain determines deformation perception on the basis of the spatial pattern of the responses of the direction-selective units. Moreover, it was shown that the mechanism underlying the deformation perception was more selective to luminance-defined than contrast-defined features.

Because the spatiotemporal motion energy did not well explain the deformation perception in our stimuli, we believe that the deformation perception is not based on a series of static deformations. Rather, it seems plausible to assume that motion mechanisms solving the aperture problem produce the kind of dynamic deformations, as reported in the previous study (Nakayama and Silverman, 1988a). Besides, in both experiments 1 and 2 of this study, the response of the direction-selective units showed the preference for downward motion when the spatial frequency of the sinusoidal deformation of a bar was high, while the spatiotemporal motion energy did not show such preference for spatiotemporal orientation involving downward motion. The downward preference is also explained in terms of solving the aperture problem (Nakayama and Silverman, 1988a). As such, rather than the spatiotemporal motion energy, the response of direction-selective units at the MT area better characterizes the perception of dynamic deformation.

Previous studies have reported that the human visual system is sensitive to direction-defined stripes (van Doorn and Koenderink, 1982a,b) and/or direction-defined gratings (Nakayama et al., 1985). Does the mechanism responsible for motion-defined structures also mediate the perception of deformation? Previous studies have consistently shown that the mechanism for the detection of a motion-defined structure is low-spatial-frequency selective. On the other hand, as shown in Figures 3*C*, 4*C*, the mechanism for deformation perception may have bandpass properties although relatively lower bands possibly contribute to the deformation perception. We, therefore, suggest that the mechanism for deformation perception is not simply equivalent to the mechanism for detecting motion-defined structures, although it is possible that some processing procedures are shared between them.

What is the possible neural mechanism for seeing deformation? It is well known that velocities are processed in the MT area. In general, velocities are captured via a large receptive field and hence processed globally (Dubner and Zeki, 1971; Newsome et al., 1985). On the other hand, some studies have reported that an identical receptive field of the MT area could locally respond to independent velocities (Majaj et al., 2007; Perrone and Krauzlis, 2008). The kind of local velocity extraction may mediate the perception of deformation, although further clarification is necessary to evaluate this possibility since the spatial properties of the local velocity extraction in the MT area are still an open issue. Structure from motion, which is occasionally involved with a complex motion structure, is also processed in the MT area (Bradley et al., 1998; Grunewald et al., 2002). There is thus a possibility that the MT area is responsible for deformation perception, which is also involved with complex motion structure.

In this study, we did not closely investigate the role of speed in the perception of dynamic deformation. Specifically, we assessed only direction parameters in the computation model, while the model proposed in previous studies (Simoncelli and Heeger, 1998) could assess both direction and speed. This was because the most revealing speed of image motion signals in our stimuli could easily anticipated for both illusory and real bar deformations, and so it was possible for us to determine the optimal speed parameter of the model in advance. On the other hand, it is known that local motion speeds determine the appearance of image deformation (Kawabe, 2018). Manipulating the speed of deformation as well as checking the speed parameters in the MT model need to be tested in future investigations.

How is the illusory deformation in our stimuli related to the footstep/inchworm illusion? In the footstep illusion (Anstis, 2001, 2004; Howe et al., 2006), an object translating at a constant velocity apparently changes its speed depending on the luminance contrast between the object and a black-white stripe background. The illusion occurs even when the contrast between the object and the background is defined by second-order features (Kitaoka and Anstis, 2015). In the inchworm illusion, a similar sort of the contrast effect on apparent speed produces the extension and contraction of the object along its motion trajectory (Anstis, 2001). The footstep/inchworm illusion is, at a glance, similar to the illusory deformation in our stimuli in terms of that the background stripe produces the change in motion appearance of a foreground object. However, there is a critical difference in the appearance between them. In the footstep illusion, the object apparently stops or reduces its speed when the contrast between the moving object and its background is low. On the other hand, in the illusion the present study reported, the static object apparently deforms when the contrast between the object and the background grating is low. That is, observers see the illusory deformation when the direction signals are produced at the intersection between, for example, a static black object and the dark part of the drifting grating. In this respect, different mechanisms basically underlie between the footstep/inchworm illusion and the illusory deformation in the present study. The footstep illusion is related to a contrast-based speed illusion and the illusory deformation in this study is related to the direction-based illusion based on the moiré pattern. According to the previous study (Anstis, 2001), the footstep illusion can also produce the two-dimensional direction illusion when the background is replaced with the two-dimensional checker-board like pattern. Anstis (2001) suggested that the apparent directional change could be predicted from the vector averaging of local motion. It is assumed that the kind of vector averaging is mediated by the processing in the MT area (Simoncelli and Heeger, 1998). Thus, the mechanism for determining direction may be common between the footstep/inchworm illusion and the illusory deformation in our study.

We acknowledge that the results of experiment 3 are explained in terms of the act of spatiotemporal filters at the first stage of our model. To detect contrast-defined features, we need to assume nonlinear processing before evaluating the spatiotemporal structure of contrast modulation. The spatiotemporal filter does not, in general, follow a nonlinear processing as like a rectification and hence it is impossible for the simple spatiotemporal filters to detect the contrast-defined structures. In experiment 3, the deformation perception was attenuated when the overall luminance of the background was equivalent to a bar. The results are consistent with the interpretation that the illusory deformation perception occurs when the spatiotemporal filters that are sensitive to luminance structures properly detect the spatiotemporal structures that eventually causes the activation of the direction selective units producing the lower spatial frequency pattern. On the other hand, we would like to emphasize that after the detection of spatiotemporal luminance structure the brain solves an aperture problem to determine global motion directions when deformation is dynamic. The solution to the aperture problem is mediated by the filters at MT. Thus, we need to assume the two-stage processing to entirely describe the dynamic deformation perception in our stimuli.

In this study, we focused on shearing deformation but not on compressive deformation. Both shearing, and compressive deformations exist in the image deformation of natural materials such as the flow of a transparent liquid. Showing that the sensitivity to the compressive deformation was higher than the sensitivity to the shearing deformation, Nakayama et al. (1985) has proposed that different mechanisms mediate the compressive deformation from the shearing deformation (see also Nakayama & Tyler, 1981). On the other hand, just how shearing and compressive deformation are processed and interact with each other in the visual system remains an open question. Psychophysical and computational investigation of simultaneous shearing and compressive deformations will lead to further understanding of how the visual system detects and interprets image deformation in natural scenes.

## Synthesis

Reviewing Editor: Li Li, New York University Shanghai Decisions are customarily a result of the Reviewing Editor and the peer reviewers coming together and discussing their recommendations until a consensus is reached. When revisions are invited, a fact-based synthesis statement explaining their decision and outlining what is needed to prepare a revision will be listed below. The following reviewer(s) agreed to reveal their identity: Szonya Durant.

Both reviewers think the ms is interesting but have concerns that should be addressed before the ms can be considered for publication. Especially, Reviewer 2 commented that the authors do not follow up on any of the interactions in the ANOVA tests, which should be addressed in the revision. I list their comments below:

Reviewer 1

This article describes a model for detecting contour deformation by investigating properties of a novel motion illusion and comparing results with a stimulus that is physically deforming. The results from the motion model are interesting, as are the behavioral results. I wonder though if the static version of the illusion needs some more consideration. As will be clear from my comments below I wasn’t quite sure whether what was being proposed was an overall deformation detector module or whether it would be more suitable for the final stage to be considered simply as an evaluation of the match between the motion outputs and deformation reports.

My 2 main questions are:

Could we have got similar results form a static version of this illusion - do the motion results not simply follow? Once we show that that the outputs from the static illusion look similar to the physically deformed version, does adding in motion not simply show that the spatio-temporal filtering works for extracting motion?

The choice of kernel weighting is based on the match with the psychophysical data. This makes sense to map the model’s modulation of responses onto the data, but it does not seem that this is a weighting the brain would have access to - this logic needs explaining a bit more

Below are all my detailed comments:

Abstract:

The results indicate that the perception of dynamic deformation is dependent on a high-level neural mechanism monitoring the spatial patterns of direction-selective units of MT areas

Suggest rather than “is dependent on” can be “well described by” as anything to do with motion has to be dependent on the spatial patterns if units in MT

Significance statement:

Why was the illusion chosen, rather than just physical deformation? This could be better motivated throughout.

Introduction

Line 46 This material non-rigidity dynamically produces image deformations of the material when the materials physically deform with time.

Awkward sentence. In the image it is contours that deform, not the material.

Line 51 Biological motion seems like an important example

Line 54 seeing -> detecting

Line 63 the pervious -> a previous

Line 67 No previous computational model does not account for the dependency of deformation perception on the spatial frequency of deformation.

Awkward phrasing, hard to follow.

Line 69 assume an additional

Line 89 need to introduce illusion and why it might be informative

Line 95 deformed ->deforming

Line 96 the perception of dynamic deformation is luminance-based, not contrast-based.

This is slightly overstated - is reliant on a difference in average luminance between inducer and object

Line 100 - suggest sticking with direction selective rather than velocity selective as velocity is direction and speed and the latter is not considered

Methods

Line 158 The temporal size and temporal wavelength

Suggest the temporal extent

Line 182 a -> α

Line 179 + 190 φ doesn’t match with symbol in equation

How were SF chosen for Experiment 2?

Results

End of Experiment : mention that the illusion did occur and summarize what ANOVA results mean: depended on orientation and SF and at each SF depended differently on orientation - does this map onto previous findings, in particular what we might expect from static?

Line 282 smaller grating orientation -> smaller grating tilt away from vertical

I found the description under Properties of units monitoring the spatial pattern of direction responses hard to follow

We evaluated how an exponential function was fitted to the psychophysical data a function of the NCC for each stimulus (Supplementary Figure 1)

Suggest here to say each point in one of the figures is the calculated NCC from on of the stimulus combo outputs shown in Figure 2

Line 309 at the specific -> at some specific

We assumed that the brain monitors all kernels with different weights and hence by using the coefficients r2 as weights

I couldn’t quite follow the logic here - the brain doesn’t have access to r2? Or are you saying that at r2 is the result of the brain’s inbuilt weighting? You are saying that there is tuning for deformations?

Exp 2 - again summarize what ANOVA means - does this tuning map onto any previous findings, in particular what we might expect from static?

Again, Exp 3 - how much is this to do with motion detection units and how much to do with the static illusion?

Discussion

I think there needs to be more in the discussion about similar static illusions.

Is there some sort of trade off here between local mechanisms looking for deformations, whilst global mechanism try to solve the aperture problems an find the overall translation of objects?

Line 456 : As shown in Figure 1, the moiré-based deformation occurs even with a static frame. On the other hand, dynamic rather than static versions of stimuli caused a stronger effect.

What do you base this claim on?

I am not clear what theses higher order units are doing? They are just assessing how much deformation is happening?

Reviewer 2

In this study, the authors examine a novel illusion in which a rigid bar appears to distort when a tilted grating drifts behind it. The authors examine the spatial frequency, orientation and luminance tuning of the illusion. The probability that observers report the illusion is higher for low spatial frequency gratings at near-vertical orientations, and when the bar is at high contrast.

They examine the pooled outputs of local direction responses along the edge of the bar and develop a set of global motion pattern templates. A weighted sum of the correlation between the template responses produces tuning functions that are similar to the psychophysical data. The authors argue that the perception of deformation is based on the responses of such mechanisms.

This illusion is interesting and the authors carefully paramaterise the tuning of the effect. The manuscript is clearly written and the experiments appear to have been conducted carefully and analyzed appropriately. The modeling involves biologically-plausible motion sensors, however the authors make many arbitrary parameter selections and they overstate the evidence that these results provide evidence for high-level neural mechanism that monitoring the spatial patterns of direction-selective units. While such mechanisms could exist, there is no published evidence that they do. The authors cannot rule out a simpler explanation that edge position changes along the edge of the bars generate motion signals. Consider the location of the edge of the bar as a grating passes behind it: the edge of the bar (defined by any edge detector) is shifted as the light and dark areas of the grating move. This position shift varies with the spatial frequency and orientation of the grating and translates along the length of the bar. This explanation does not require any integration or high-level neural mechanism and is consistent with the liminance tuning of the illusion.

The illusion is similar to the Inchworm illusion of Anstis (2001), which should be cited. The explanation advanced by Anstis is based on local contrast effects that would account for the present illusion.

Specific Comments

The argument that the perception of dynamic deformation is based on the spatial modulation of direction responses is tautological.

Please define the normalisation stage in the model.

67 ‘No previous computational model does not account for the"

-> No previous computational model accounts for the

69 ‘Hence, it is necessary to assume’ AN ‘additional mechanism’

70.“ Moreover, it remained unclear” -> Moreover, it remains unclear

401 “Moreover, the proportion for 76 cd/m2 was the chance level.”

50% is not chance in a Yes/No task, it means that there was visible distortion reported on around 50% trials or subjects

Reference

Anstis, S. M. (2001). Footsteps and inchworms: Illusions show that contrast modulates motion salience. Perception, 30, 785-794.

## Author Response

Synthesis Statement for Author (Required):

Both reviewers think the ms is interesting but have concerns that should be addressed before the ms can be considered for publication. Especially, Reviewer 2 commented that the authors do not follow up on any of the interactions in the ANOVA tests, which should be addressed in the revision. I list their comments below:

[Reply from the authors]

Thank you very much for the careful reviews by Reviewers 1 and 2. As one of the reviewers pointed out, the discussion about the interaction in the ANOVA test was not possibly sufficient. In the revised manuscript, we added some descriptions to discuss the interaction in the ANOVA test.

-------------------------------------------------------------------------------------------------------------------------------[Comment by Reviewer 1]

Reviewer 1

This article describes a model for detecting contour deformation by investigating properties of a novel motion illusion and comparing results with a stimulus that is physically deforming. The results from the motion model are interesting, as are the behavioral results. I wonder though if the static version of the illusion needs some more consideration. As will be clear from my comments below I wasn’t quite sure whether what was being proposed was an overall deformation detector module or whether it would be more suitable for the final stage to be considered simply as an evaluation of the match between the motion outputs and deformation reports.

[Reply from the authors]

We really appreciate your careful review of the manuscript. We think what we propose is not necessarily deformation detector modules. As Reviewer 1 suggested, we need to carefully discuss the role of the final stage in our model. The final stage in principle can detect various bands of the spatial frequency of direction-selective responses. In this respect, our previous suggestion that the units in the final stage are sensitive to the specific range of the spatial frequency of direction-selective units responses was inaccurate. In the revised manuscript, we carefully discussed that the observers used the output of the unit to determine whether the stimuli contained deformation or not.

-------------------------------------------------------------------------------------------------------------------------------

[Comment by Reviewer 1]

My 2 main questions are:

Could we have got similar results from a static version of this illusion - do the motion results not simply follow? Once we show that that the outputs from the static illusion look similar to the physically deformed version, does adding in motion not simply show that the spatio-temporal filtering works for extracting motion?

[Reply from the authors]

Thank you very much for giving us an opportunity to clarify this issue. We believe that the perception of dynamic deformation is not just identical to the perception of static deformation in succession. We have added descriptions to explain our idea to the fourth paragraph of the General Discussion part.

-------------------------------------------------------------------------------------------------------------------------------

[Comment by Reviewer 1]

The choice of kernel weighting is based on the match with the psychophysical data. This makes sense to map the model’s modulation of responses onto the data, but it does not seem that this is a weighting the brain would have access to - this logic needs explaining a bit more

[Reply from the authors]

Thank you very much for pointing this out. As Reviewer 1 suggested, we acknowledge that the logic for using r2s as weights to modulate the output of the higher-order unit was unclear in the previous manuscript. To the second paragraph of “Properties of units monitoring the spatial pattern of direction responses” section, we have added several descriptions in order to make this point clearer.

-------------------------------------------------------------------------------------------------------------------------------

[Comment by Reviewer 1]

Below are all my detailed comments:

Abstract:

The results indicate that the perception of dynamic deformation is dependent on a high-level neural mechanism monitoring the spatial patterns of direction-selective units of MT areas

Suggest rather than “is dependent on” can be “well described by” as anything to do with motion has to be dependent on the spatial patterns if units in MT

[Reply from the authors]

Thank you very much for your suggestion. We employed the phrase you suggested accordingly because we agree that the phrase was more suitable here than the previous one.

--------------------------------------------------------------------------------------

[Comment by Reviewer 1]

Significance statement:

Why was the illusion chosen, rather than just physical deformation? This could be better motivated throughout.

[Reply from the authors]

We agree with Reviewer 1 that the reason why we used an illusory deformation was unclear. To several places in the revised manuscript, we have added descriptions to make the reason clearer.

---------------------------------------------------------------------------------------

[Comment by Reviewer 1]

Introduction

Line 46 This material non-rigidity dynamically produces image deformations of the material when the materials physically deform with time. Awkward sentence. In the image it is contours that deform, not the material.

[Reply from the authors]

We agree with Reviewer 1 and have added a phrase to say that both contour and texture deform in the retinal image.

---------------------------------------------------------------------------------------

[Comment by Reviewer 1]

Line 51 Biological motion seems like an important example

[Reply from the authors]

We have cited several studies on biological motion here.

---------------------------------------------------------------------------------------

[Comment by Reviewer 1]

Line 54 seeing -> detecting

[Reply from the authors]

Fixed.

---------------------------------------------------------------------------------------

[Comment by Reviewer 1]

Line 63 the pervious -> a previous

[Reply from the authors]

Fixed.

---------------------------------------------------------------------------------------

[Comment by Reviewer 1]

Line 67 No previous computational model does not account for the dependency of deformation perception on the spatial frequency of deformation.

Awkward phrasing, hard to follow.

[Reply from the authors]

Thank Reviewer 1 for pointing this out. We have modified this part by removing ‘does not’ from the sentence.

---------------------------------------------------------------------------------------

[Comment by Reviewer 1]

Line 69 assume an additional

[Reply from the authors]

We rewrote here in the following; ‘Hence, additional examinations are necessary to fully understand deformation perception in human observers.’

---------------------------------------------------------------------------------------

[Comment by Reviewer 1]

Line 89 need to introduce illusion and why it might be informative

[Reply from the authors]

We changed the order of Experiment 1 and Experiment 2 in the revised manuscript because it is more comprehensive with this order than the previous one why we conducted an experiment with illusory deformation display. Upon this change, to the final paragraph of Introduction, we have added several descriptions in order to make it clearer how we used the illusory deformation display.

---------------------------------------------------------------------------------------

[Comment by Reviewer 1]

Line 95 deformed ->deforming

[Reply from the authors]

Fixed.

---------------------------------------------------------------------------------------

[Comment by Reviewer 1]

Line 96 the perception of dynamic deformation is luminance-based, not contrast-based.

This is slightly overstated - is reliant on a difference in average luminance between inducer and object

[Reply from the authors]

We rewrote this part in the following ‘In Experiment 3, we show that the perception of illusory dynamic deformation is attenuated when the average luminance of background grating is equivalent to the luminance of a bar' (at the last paragraph of Introduction section)

---------------------------------------------------------------------------------------

[Comment by Reviewer 1]

Line 100 - suggest sticking with direction-selective rather than velocity selective as velocity is direction and speed and the latter is not considered

[Reply from the authors]

Reviewer 1’s criticism is correct. We used the term ‘direction-selective’ throughout the manuscript.

---------------------------------------------------------------------------------------

[Comment by Reviewer 1]

Methods

Line 158 The temporal size and temporal wavelength

Suggest the temporal extent

[Reply from the authors]

We have modified this part in the following: ‘Both the temporal size and temporal wavelength of the filters were kept constant at 30 frames (0.5 seconds)’. We chose a spatiotemporal filter with a relatively large temporal extent because the expected speed in stimuli is slow while the spatial frequency of stimuli was relatively high. Moreover, the temporal filter having the temporal size of 30 frames was expected to be able to well capture motion signals that were created by the half cycle of stimuli; the full cycle of stimuli involved a round-trip motion. Because the round-trip motion was not desirable for our analysis, we needed to focus on a unidirectional motion signal generated by the half-cycle of stimuli.

---------------------------------------------------------------------------------------

[Comment by Reviewer 1]

Line 182 a -> α

[Reply from the authors]

Fixed.

---------------------------------------------------------------------------------------

[Comment by Reviewer 1]

Line 179 + 190 φ doesn’t match with symbol in equation

[Reply from the authors]

Fixed.

---------------------------------------------------------------------------------------

[Comment by Reviewer 1]

How were SF chosen for Experiment 2?

[Reply from the authors]

To the Method part of Experiment 1 (i.e., previously Experiment 2), we have added a description to explain that we chose the range of spatial frequency of deformation to cover the range tested in the previous study.

---------------------------------------------------------------------------------------

[Comment by Reviewer 1]

Results

End of Experiment : mention that the illusion did occur and summarize what ANOVA results mean: depended on orientation and SF and at each SF depended differently on orientation - does this map onto previous findings, in particular what we might expect from static?

[Reply from the authors]

We have added some discussions to interpret ANOVA results at the end of each experiment. As replied to the previous comments by Reviewer 1, the appearance of dynamic image deformation included some properties that cannot be explained by the successive presentation of static image deformation. For example, the perceived motion direction of deforming contour is well consistent with the interpretation that an aperture problem is solved to determine perceived motion direction. Moreover, as the previous study (Nakayama & Silverman, 1985) also showed, when the spatial frequency of deformation was high, the observers saw the rigid translation of wavy patterns along the edge of the bar. To explain both of the two appearances of dynamic image deformation, the model for the static deformation is insufficient. For these reasons, we continued to discuss our results in terms of dynamic rather than static deformation perception.

---------------------------------------------------------------------------------------

[Comment by Reviewer 1]

Line 282 smaller grating orientation -> smaller grating tilt away from vertical

[Reply from the authors]

Fixed.

---------------------------------------------------------------------------------------

[Comment by Reviewer 1]

I found the description under Properties of units monitoring the spatial pattern of direction responses hard to follow

We evaluated how an exponential function was fitted to the psychophysical data a function of the NCC for each stimulus (Supplementary Figure 1)

Suggest here to say each point in one of the figures is the calculated NCC from one of the stimulus combo outputs shown in Figure 2

[Reply from the authors]

We have rewritten this part and also added Figure 3b (previously Supplementary Figure 1) in order to make our method clearer.

---------------------------------------------------------------------------------------

[Comment by Reviewer 1]

Line 309 at the specific -> at some specific

[Reply from the authors]

Fixed.

---------------------------------------------------------------------------------------

[Comment by Reviewer 1]

We assumed that the brain monitors all kernels with different weights and hence by using the coefficients r2 as weights

I couldn’t quite follow the logic here - the brain doesn’t have access to r2? Or are you saying that at r2 is the result of the brain’s inbuilt weighting? You are saying that there is tuning for deformations?

[Reply from the authors]

We agree with Reviewer 1 that our previous manuscript lacked descriptions sufficiently explaining why we used r2 to weigh the output of higher-order units depending on the spatial frequency of deformation. We have added descriptions to explain this to the section ‘Properties of units monitoring the spatial pattern of direction responses in the Results & Discussion sections.

---------------------------------------------------------------------------------------

[Comment by Reviewer 1]

Exp 2 - again summarize what ANOVA means - does this tuning map onto any previous findings , in particular what we might expect from static?

[Reply from the authors]

We have added some descriptions to interpret the ANOVA results. For the reasons described above, we continue to interpret our results in terms of dynamic rather than static deformation perception.

---------------------------------------------------------------------------------------

[Comment by Reviewer 1]

Again, Exp 3 - how much is this to do with motion detection units and how much to do with the static illusion?

[Reply from the authors]

For this issue, we acknowledge that the locus of the luminance effects on deformation perception possibly occurred at V1 rather than MT. Hence, to General discussion, we have added descriptions to supplement our idea that early filters that are possibly located at V1 are sensitive to spatiotemporal luminance flows that are defined mainly by luminance and the output of the early filters are assessed at the MT filter to solve an aperture problem.

---------------------------------------------------------------------------------------

[Comment by Reviewer 1]

Discussion

I think there needs to be more in the discussion about similar static illusions.

[Reply from the authors]

We agree with Reviewer 1 and thus have added descriptions to explain the role of static deformations in the results of the present study to the fourth paragraph of General Discussion.

---------------------------------------------------------------------------------------

[Comment by Reviewer 1]

Is there some sort of trade off here between local mechanisms looking for deformations, whilst global mechanism try to solve the aperture problems an find the overall translation of objects?

[Reply from the authors]

We have discussed this issue in the fourth paragraph of the General Discussion.

---------------------------------------------------------------------------------------

[Comment by Reviewer 1]

Line 456 : As shown in Figure 1, the moiré-based deformation occurs even with a static frame. On the other hand, dynamic rather than static versions of stimuli caused a stronger effect. What do you base this claim on?

[Reply from the authors]

As Reviewer 1 suspected, this description seemed to lack theoretical rationale. We have omitted a paragraph including this sentence because the suggestion in the paragraph has not been supported by the results of the present study.

---------------------------------------------------------------------------------------

[Comment by Reviewer 1]

I am not clear what theses higher order units are doing? They are just assessing how much deformation is happening?

[Reply from the authors]

We think that the brain determines whether stimuli contain deformation or not by using the output of the higher-order units that represent the spatial pattern of the responses of the direction-selective units. We have rewritten the manuscript to make this point clearer.

---------------------------------------------------------------------------------------

[Comment by Reviewer 2]

Reviewer 2

In this study, the authors examine a novel illusion in which a rigid bar appears to distort when a tilted grating drifts behind it. The authors examine the spatial frequency, orientation and luminance tuning of the illusion. The probability that observers report the illusion is higher for low spatial frequency gratings at near-vertical orientations, and when the bar is at high contrast.

They examine the pooled outputs of local direction responses along the edge of the bar and develop a set of global motion pattern templates. A weighted sum of the correlation between the template responses produces tuning functions that are similar to the psychophysical data. The authors argue that the perception of deformation is based on the responses of such mechanisms.

This illusion is interesting and the authors carefully paramaterise the tuning of the effect. The manuscript is clearly written and the experiments appear to have been conducted carefully and analyzed appropriately. The modeling involves biologically-plausible motion sensors, however the authors make many arbitrary parameter selections and they overstate the evidence that these results provide evidence for high-level neural mechanism that monitoring the spatial patterns of direction-selective units. While such mechanisms could exist, there is no published evidence that they do. The authors cannot rule out a simpler explanation that edge position changes along the edge of the bars generate motion signals. Consider the location of the edge of the bar as a grating passes behind it: the edge of the bar (defined by any edge detector) is shifted as the light and dark areas of the grating move. This position shift varies with the spatial frequency and orientation of the grating and translates along the length of the bar. This explanation does not require any integration or high-level neural mechanism and is consistent with the liminance tuning of the illusion.

The illusion is similar to the Inchworm illusion of Anstis (2001), which should be cited. The explanation advanced by Anstis is based on local contrast effects that would account for the present illusion.

Reference

Anstis, S. M. (2001). Footsteps and inchworms: Illusions show that contrast modulates motion salience. Perception, 30, 785-794.

[Reply from the authors]

Thank Reviewer 2 very much for the careful review of the manuscript. We also appreciate that Reviewer 2 directed our attention to Anstis’s footstep illusion and inchworm effect. On the other hand, we believe that the appearance of our illusion is not entirely explained by the mechanism for other illusions the previous studies reported. We have added descriptions for the explanation of this point to the fourth paragraph of the General Discussion.

---------------------------------------------------------------------------------------

[Comment by Reviewer 2]

Specific Comments

The argument that the perception of dynamic deformation is based on the spatial modulation of direction responses is tautological.

[Reply from the authors]

We agree with Reviewer 2’s suggestion and hence tried to remove such tautological expressions from the manuscript.

---------------------------------------------------------------------------------------

[Comment by Reviewer 2]

Please define the normalisation stage in the model.

[Reply from the authors]

We have defined the normalization stage in the Method section.

---------------------------------------------------------------------------------------

[Comment by Reviewer 2]

67 ‘No previous computational model does not account for the”

-> No previous computational model accounts for the

[Reply from the authors]

Fixed.

---------------------------------------------------------------------------------------

[Comment by Reviewer 2]

69 “Hence, it is necessary to assume’ AN’ additional mechanism”

[Reply from the authors]

In the course of the revision, this sentence has been removed.

---------------------------------------------------------------------------------------

[Comment by Reviewer 2]

70.” Moreover, it remained unclear” -> Moreover, it remains unclear

[Reply from the authors]

Fixed.

---------------------------------------------------------------------------------------

[Comment by Reviewer 2]

401 “Moreover, the proportion for 76 cd/m2 was the chance level.”

50% is not chance in a Yes/No task, it means that there was visible distortion reported on around 50% trials or subjects

[Reply from the authors]

We agree with Reviewer 2 and removed this sentence accordingly.
